# Characterization of the Sorbitol Utilization Cluster of the Probiotic *Pediococcus parvulus* 2.6: Genetic, Functional and Complementation Studies in Heterologous Hosts

**DOI:** 10.3389/fmicb.2017.02393

**Published:** 2017-12-05

**Authors:** Adrian Pérez-Ramos, Maria L. Werning, Alicia Prieto, Pasquale Russo, Giuseppe Spano, Mari L. Mohedano, Paloma López

**Affiliations:** ^1^Biological Research Center (CIB), Consejo Superior de Investigaciones Científicas, Madrid, Spain; ^2^Center of Research and Transfer of Catamarca (CITCA), Consejo Nacional de Investigaciones Científicas y Técnicas, Catamarca, Argentina; ^3^Department of Agricultural, Food and Environmental Sciences, University of Foggia, Foggia, Italy

**Keywords:** *Pediococcus parvulus*, exopolysaccharides, β-glucans, sorbitol, lactic acid bacteria, probiotic

## Abstract

*Pediococcus parvulus* 2.6 secretes a 2-substituted (1,3)-β-D-glucan with prebiotic and immunomodulatory properties. It is synthesized by the GTF glycosyltransferase using UDP-glucose as substrate. Analysis of the *P. parvulus* 2.6 draft genome revealed the existence of a sorbitol utilization cluster of six genes (*gutFRMCBA*), whose products should be involved in sorbitol utilization and could generate substrates for UDP-glucose synthesis. Southern blot hybridization analysis showed that the cluster is located in a plasmid. Analysis of metabolic fluxes and production of the exopolysaccharide revealed that: (i) *P. parvulus* 2.6 is able to metabolize sorbitol, (ii) sorbitol utilization is repressed in the presence of glucose and (iii) sorbitol supports the synthesis of 2-substituted (1,3)-β-D-glucan. The sorbitol cluster encodes two putative regulators, GutR and GutM, in addition to a phosphoenolpyruvate-dependent phosphotransferase transport system and sorbitol-6-phosphate dehydrogenase. Therefore, we investigated the involvement of GutR and GutM in the expression of *gutFRMCBA*. The promoter-probe vector pRCR based on the *mrfp* gene, which encodes the fluorescence protein mCherry, was used to test the potential promoter of the cluster (P*_gut_*) and the genes encoding the regulators. This was performed by transferring by electrotransformation the recombinant plasmids into two hosts, which metabolize sorbitol: *Lactobacillus plantarum* and *Lactobacillus casei*. Upon growth in the presence of sorbitol, but not of glucose, only the presence of P*_gut_* was required to support expression of *mrfp* in *L. plantarum*. In *L. casei* the presence of sorbitol in the growth medium and the pediococcal *gutR* or *gutR* plus *gutM* in the genome was required *for* P*_gut_* functionality. This demonstrates that: (i) P*_gut_* is required for expression of the *gut* cluster, (ii) P*_gut_* is subjected to catabolic repression in lactobacilli, (iii) GutR is an activator, and (iv) in the presence of sorbitol, *trans*-complementation for activation of P*_gut_* exists in *L. plantarum* but not in *L. casei*.

## Introduction

Sorbitol, also named D-glucitol, is a six-carbon sugar polyol widespread in plants, particularly in fruits, such as berries, cherries, plums, pears and apples. However, sorbitol is obtained industrially, by catalytic hydrogenation of glucose or glucose/fructose mixtures. This polyol has a relative sweetness of about 60% compared to that of sucrose, high-water solubility and is largely used as a low calorie sweetener, humectant, texturizer and softener (Zumbé et al., 2001). In addition, sorbitol is used in the production of pharmaceutical compounds, such as sorbose and ascorbic acid, and as a vehicle for drug-suspension (Silveira and Jonas, 2002). Sorbitol has also a potential prebiotic effect *in vivo*, since it does not contribute to the formation of dental caries, is slowly and only partially absorbed in the small intestine and can reach the colon where it can act as substrate for bacterial fermentation. Supplementation with sorbitol resulted in enrichment of lactobacilli in rat colon and cecum (Sarmiento-Rubiano et al., 2007).

Sorbitol absorption is mediated by dose and concentration. Doses greater than 30 g can cause water retention, resulting in osmotic diarrhea, bloating, flatulence, cramping and abdominal pain (Fernández-Bañares et al., 2009). These doses vary depending on the condition of the intestinal absorption surface. In patients with malabsorption, the ingestion of 5–20 g, provoked diarrhea and gastrointestinal complications (Montalto et al., 2013). In the colon, this sugar alcohol is metabolized by some species of *Lactobacillus* and is also a preferred carbon source for human intestinal bifidobacteria (Sarmiento-Rubiano et al., 2007).

Furthermore, utilization of sorbitol as a carbon source has been described in a variety of bacteria within the fila proteobacteria (Yamada and Saier, 1988; Aldridge et al., 1997) and firmicutes (Tangney et al., 1998; Boyd et al., 2000; Yebra and Pérez-Martínez, 2002). Among the firmicutes, there are some lactic acid bacteria (LAB) with catabolic pathways for sorbitol metabolism (Rhodes and Kator, 1999; Sarmiento-Rubiano et al., 2007). These pathways are encoded by genes organized in *gut* operons, and include the sorbitol transport system, sorbitol-6-phosphate dehydrogenase (S6PD) as well as regulatory protein(s), and those of *Lactobacillus casei* and *Lactobacillus plantarum* have been characterized (Nissen et al., 2005; Ladero et al., 2007; Alcantara et al., 2008).

Sorbitol is transported into the cells and phosphorylated to sorbitol-6-phosphate by a phosphopyruvate-dependent phosphotransferase (PTS) sorbitol system (PTS^gut^). Each PTS is composed of two cytoplasmic enzymes, common to the transport of different compounds (EI and HPr) and of different membrane-associated enzyme complexes (EII), specific for one, or several substrates. The genes *gutC*, *gutB* and *gutA* encode the EII domain of a sorbitol PTS (Alcantara et al., 2008). The *gutF* gene encodes a sorbitol-6-P dehydrogenase, which catalyzes the conversion of sorbitol-6-phosphate to fructose-6-phosphate, a compound that is introduced into the glycolytic pathway with NADH regeneration (Nissen et al., 2005). The *gutR* and *gutM* genes encode two regulatory proteins. The role of the GutM and GutR proteins has been studied in *Escherichia coli*, operating GutM as an activator and GutR as a repressor (Yamada and Saier, 1988). In the firmicutes group, the analyzed *gut* operons contain homologs to the *gutM* and *gutR* genes, but the role of GutR regulator is different from that of *E. coli*. The GutR of *L. casei* has been functionally characterized and it has been shown to be a PTS-controlled transcriptional activator, via a PTS regulation binding domain (PRD) (Stülke et al., 1998). Also, both the GutR binding sequence and the PRD domain are conserved in firmicutes. GutM encodes a highly conserved protein in firmicutes and in *L. casei* plays a regulatory role (Alcantara et al., 2008).

*Pediococcus parvulus* 2.6 (Werning et al., 2006) (previously named *Pediococcus damnosus*) is a lactic acid bacteria isolated from a ropy cider (Fernández et al., 1995). This LAB produces a 2-substituted (1,3)-β-D-glucan exopolysaccharide (EPS) (Dueñas-Chasco et al., 1997), with high molecular mass (>10^6^ Da), and whose rheological properties showed its potential utility as a biothickening agent (Velasco et al., 2009). The presence of this EPS improves some probiotic features of *P. parvulus* 2.6, including tolerance to simulated gastrointestinal conditions and adherence to Caco-2 cell lines and reduces inflammation-related cytokine levels produced by polarized macrophages (Fernández de Palencia et al., 2009; Immerstrand et al., 2010). Moreover, the purified EPS improves the growth, viability and adhesion capability of probiotic microorganisms (Russo et al., 2012), also it activates macrophages with anti-inflammatory effects (Notararigo et al., 2014), and decreases the levels of the proinflammatory IL8 in human intestine cultures (Notararigo et al., unpublished data). The draft genome of *P. parvulus* 2.6 has been determined (Pérez-Ramos et al., 2016), and its analysis showed the existence of a putative sorbitol utilization *gut* operon in this bacterium. Thus, this current work focuses on the genomic location, expression and metabolic involvement of the *gut* operon of *P. parvulus* 2.6 in sorbitol catabolism, as well as its interplay with EPS production by this bacterium.

## Materials and Methods

### Bacterial Strains and Growth Conditions

The bacteria used in this work are listed in **Table 1**. *Pediococcus* and *Lactobacillus* strains were routinely grown in de Man Rogosa Sharpe (MRS) broth (Pronadisa, Madrid, Spain) at 30°C and 37°C, respectively. *Lactococcus lactis* strains were grown in ESTY broth (Pronadisa) supplemented with 0.5% glucose at 30°C. When bacteria carried the pRCR plasmid or its derivatives the medium was supplemented with chloramphenicol (Cm) at 5 μg mL^-1^ for *L. lactis* and at 10 μg mL^-1^ for lactobacilli. *E. coli* V517 was grown in LB broth and incubated at 37°C.

**Table 1:** Bacteria used in this work.

Bacteria	Plasmid	Resistance	Characteristics	Reference
*Pediococcus parvulus* 2.6	pPP1, pPP2, and pPP3	–	2-substituted (1,3)-β-D-glucan producer	Pérez-Ramos et al., 2016
*P. parvulus* 2.6NR	pPP1 and pPP3	–	Non-EPS-producing strain. Derivative of 2.6 strain by pPP2 plasmid curing	Fernández et al., 1995
*Lactococcus lactis* subsp. *cremoris* MG1363	–	–	Plasmid free type strain used for plasmid cloning	Wegmann et al., 2007
*L. lactis* subsp. *cremoris* MG1363[pRCR]	pRCR	Cm^R^	Source of promoter probe pRCR containing the *mrfp* gene, which encodes the fluorescent mCherry protein	Mohedano et al., 2015
*Escherichia coli* V517	8 plasmids pVA517A through pVA517H	ND	Source of plasmids used as references in agarose gel analysis	Macrina et al., 1978
*Lactobacillus casei* BL23	–	–	Bacteria used for heterologous gene expression	Mazé et al., 2010
*L. casei* BL23[pRCR16]	[pRCR16]	Cm^R^	Derivative of pRCR by cloning of P*_gut_* upstream of *mrfp*	This study
*L. casei* BL23[pRCR17]	[pRCR17]	Cm^R^	Derivative of pRCR16 by cloning of *gutR* upstream of *mrfp*	This study
*L. casei* BL23[pRCR18]	[pRCR18]	Cm^R^	Derivative of pRCR16 by cloning of *gutM* upstream of *mrfp*	This study
*L. casei* BL23[pRCR19]	[pRCR19]	Cm^R^	Derivative of pRCR16 by cloning of *gutMR* upstream of *mrfp*	This study
*Lactobacillus plantarum* 90	1 uncharacterized plasmid	ND	Bacteria used for heterologous gene expression	Lamontanara et al., 2015
*L. plantarum* 90[pRCR16]	[pRCR16]	Cm^R^	Derivative of pRCR by cloning of P*_gut_* upstream of *mrfp*	This study
*L. plantarum* 90[pRCR17]	[pRCR17]	Cm^R^	Derivative of pRCR16 by cloning of *gutR* upstream of *mrfp*	This study
*L. plantarum* 90[pRCR18]	[pRCR18]	Cm^R^	Derivative of pRCR16 by cloning of *gutM* upstream of *mrfp*	This study
*L. plantarum* 90[pRCR19]	[pRCR19]	Cm^R^	Derivative of pRCR16 by cloning of *gutMR* upstream of *mrfp*	This study

*ND, no determined; Cm^*R*^, resistance to chloramphenicol*.

For evaluation of sorbitol utilization, *P. parvulus* strains were grown in a MRS broth made by components (de Man et al., 1960) without glucose, pH was adjusted to 5.2 and the medium supplemented with 10 mM glucose (MRSG), 30 mM sorbitol (MRSS) or 10 mM glucose plus 30 mM sorbitol (MRSGS) at 30°C. Prior selection of conditions for growth in presence of sorbitol several tests were performed. First various carbon sources were tested (10 mM glucose, 10 mM fructose or 10 mM maltose) and pH at 6.8, 5.2 or 4.0 and then influence of aeration was evaluated in presence of 10 mM glucose at either pH 6.8 and 5.2 (results not show).

For evaluation of mCherry expression, *Lactobacillus* strains were grown in a MRSG containing 55 mM (1% w/v) glucose or in a MRSS containing 55 mM (1% w/v) sorbitol at 37°C.

### Plasmidic DNA Preparations

Total plasmidic DNA preparations of *P. parvulus* 2.6 and 2.6NR strains were prepared as follows. Bacterial cultures were grown to an optical density at 600 nm (OD_600__nm_) of 2.5, and 100 mL of each culture were sedimented by centrifugation at 10,000 × *g* for 20 min at 4°C. The cells were resuspended in 4 mL of a solution containing 50 mM Tris/HCl pH 8.0, 10 mM EDTA, lysozyme (30 mg mL^-1^) and RNasa A (10 μg mL^-1^), and incubated for 30 min at 37°C. Then, 4 mL of a solution containing 220 mM NaOH and 1.33% sodium dodecyl sulfate) were added and samples were incubated for 5 min at room temperature. Upon addition of 5 M potassium acetate pH 5.0 (4 mL), samples were centrifugated at 10,000 × *g* for 15 min at 21°C. The DNA present in the supernatants was precipitated, concentrated by addition of 8.7 mL of isopropanol, sedimented by centrifugation at 10,000 × *g* for 15 min at 4°C, and resuspended in 10 mM Tris, 1 mM EDTA buffer (4.3 mL). The DNA preparation was deproteinated by treatment with 7.5 M ammonium acetate (2.7 mL) and phenol (4.3 mL) during 5 min at room temperature and then sedimented at 10,000 × *g* for 5 min at 21°C. The aqueous phase containing total plasmidic DNA was further purified by isopycnic CsCl density gradient centrifugation and dialysis as previously described (López et al., 1989). The final recovery was 54 μg and 58 μg for 2.6 and 2.6NR DNA preparations, respectively.

The recombinant plasmids from the lactococcal and lactobacilli strains were isolated using the High pure plasmid isolation kit (Roche) as follows. Bacteria were grown until stationary phase (10^9^ colony forming units mL^-1^) and 1 mL of each culture were sedimented by centrifugation at 10,000 × *g* for 10 min at 4°C. Cells were resuspended in solution I of the kit supplemented with lysozyme (30 mg mL^-1^) and were incubated for 30 min at 37°C. Then, plasmid isolation were performed as described in the kit protocol, eluting the plasmidic DNA in 100 μL at approximately 100 ng μL^-1^.

### Sequencing

DNA sequencing was performed by the dideoxy method at Secugen (Madrid, Spain). The sequencing of the sorbitol utilization cluster and the flanking regions of pPP1 of *P. parvulus* 2.6 was performed using total plasmidic DNA preparations of the bacterium (see above) with the walking strategy and the sequence has been deposited in GenBank (accession No MF766019). The lack of sorbitol cluster in the 2.6NR strain was confirmed by sequencing of its pPP1 plasmid by using as substrates a total plasmidic preparation of 2.6NR strain and either pPP1^∗^F or pPP1^∗^R primers (see **Table 2**) In addition, in the case of sequencing with pPP1^∗^F, it was also used as substrate the product of a polymerization reaction catalyzed by the bacteriophage Φ29 DNA polymerase with plasmidic DNA of *P. parvulus* 2.6NR and hexamers containing random sequences.

**Table 2:** Oligonucleotides used in this work.

Primers	Sequence (5′- 3′)	Utilization	Amplicon size (bp)
pts1F	TGCGGAAGCGGTTAATCGGCT	*gutF* DNA probe^a^	618
pts1R	CCACGACTCTTGCCTCCCGCA		
ptsRF	CGAACTGGAAGCAACCTGGGA	*gutR* DNA probe^a^	652
ptsRR	CCGATGAATAATTGGCGCTGC		
ptsBF	GGAATGGAAGCTGTTGATGGC	*gutB* DNA probe^a^	643
ptsBR	CAACGCCAATCAAGGTCCCGA		
pgutBglIIF	GAAGATCTACCATATGGCGATAATGAAAA	Cloning of P*_gut_* in pRCR^a^	186
pgutXmaIR	TCGCTCCCGGGTCATTTCTTTTC		
gutRXmaIF	CGTGGTTAACCCGGGAATTTTAGTTG	Cloning of *gutR* in pRCR16^a^	1952
gutRXbaIR	GCTCTAGAAACGCACTGACTAGGATCA		
gutMXmaIF	TCCCCCCGGGTTAAATCAGTTGATGGA	Cloning of *gutM* in pRCR16^a^	597
gutMXbaIR	GCTCTAGAACAGCCCATAAAGCCC		
gutRXmaIF	CGTGGTTAACCCGGGAATTTTAGTTG	Cloning of *gutRM* in pRCR16^a^	2472
gutMXbaIR	GCTCTAGAACAGCCCATAAAGCCC		
pPP1^∗^F	CATAGTTCACTGGGCTACCA	Sequencing of pPP1^∗b^	–
pPP1^∗^R	TAGCGGTGCCTCCCTTTAAT		–

^a^*Plasmidic DNA preparations of P. parvulus 2.6 was used as substrate for the PCR reactions. ^b^Plasmidic DNA preparations of P. parvulus 2.6NR was used as substrate for the DNA sequencing*.

### Construction of pRCR16, pRCR17, pRCR18, and pRCR19

**FIGURE 1:**
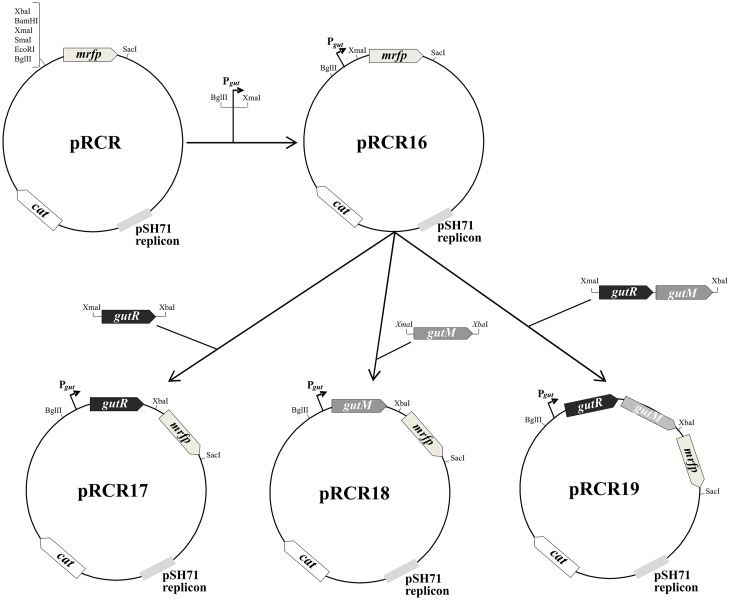
Scheme of construction of plasmids pRCR16, pRCR17, pRCR18, and pRCR19. Maps of these plasmids and of the parental pRCR are depicted. The pertinent restriction sites are shown, as well as the putative promoter of the *gut* operon (P*_gut_*) and the following genes: *mrfp, cat*, *gutR* and *gutM* encoding the mCherry, a chloramphenicol acetyltransferase, GutR and GutM, respectively.

A region located upstream of the *P. parvulus* 2.6 *gut* operon carrying the putative P*_gut_* promoter and the *gutR* and *gutM* genes was cloned into the promoter probe pRCR vector. To this end, three DNA regions of pPP1 plasmid were amplified with Phusion High Fidelity Polymerase (PHFP, ThermoFisher Scientific) by using a plasmidic DNA preparation of *P. parvulus* 2.6 and the primers depicted in **Table 2**, which have homology with pPP1 DNA and carry restriction sites suitable for cloning. Plasmid pRCR16 (**Figure 1**) was generated by ligation of the P*_gut_* promoter to the pRCR promoter probe vector (Mohedano et al., 2015), after double digestion of both DNAs with BglII and XmaI (New England Biolabs, Ipswich, MA, United States), with the T4 DNA ligase (New England Biolabs). Then, between the XmaI and XbaI restriction sites of pRCR16 three amplicons were independently cloned, containing *gutR*, *gutM* or *gutRM*, generating plasmids pRCR17, pRCR18 and pRCR19, respectively. The clonings were performed in *L. lactis* MG1363, the ligations mixtures were used to transform the bacteria by electroporation (25 μF, 2.5 kV and 200 Ω in 0.2 cm cuvettes), as previously described (Dornan and Collins, 1987) and transformants were selected in ESTY-agar plates supplemented with Cm at 5 μg mL^-1^. The inserts present in the new four recombinant plasmids were confirmed by automated sequencing. Then, DNA preparations of pRCR17, pRCR18 and pRCR19 obtained from *L. lactis* MG1363 (0.5 μg) were used for transfer to lactobacilli by electroporation (25 μF, 1.3 kV and 200 Ω in 0.1 cm cuvettes) as previously described (Berthier et al., 1996) and transformants were selected in MRSG-agar plates supplemented with Cm at 10 μg mL^-1^.

### Southern Hybridization

Plasmid samples were fractionated by electrophoresis in a 0.7% agarose gel and DNA molecules were revealed by staining with ethidium bromide at 0.5 μg mL^-1^. The image of the gels was obtained with GelDoc 200 (BioRad) and the bands were quantitated with the Quantity One 4.5.2 software (BioRad). The DNA fragments were transferred to a nylon membrane Biodyne A (PALL Gelman Laboratory, AnnArbor, MI, United States) by 5 inches Hg of vacuum for 2 h using the Vacuum Blotter model 785 (Bio-Rad). Internal regions of *gutF*, *gutR* and *gutB* genes were amplified by PCR generating amplicons 1, 2, and 3, respectively, in reactions catalyzed by PHFP, and by using as substrate total plasmidic DNA preparation of *P. parvulus* 2.6 and the primer pairs shown in **Table 2**. Then, the amplicons were labeled with digoxigenin-dUTP by using the DIG high prime DNA labeling and detection starter kit II (Roche, Mannheim, Germany). Each DIG-labeled DNA probe (25 ng mL^-1^) was used for hybridization at 45°C following the specifications of the kit’s supplier. The hybridization bands were revealed with the chemiluminescent substrate CSPD, and the signals were detected with the LAS-3000 imaging system (Fujifilm, Stamford, CT, United States).

### Analysis of the Metabolic Fluxes of *P. parvulus* and Its EPS Production

**FIGURE 2:**
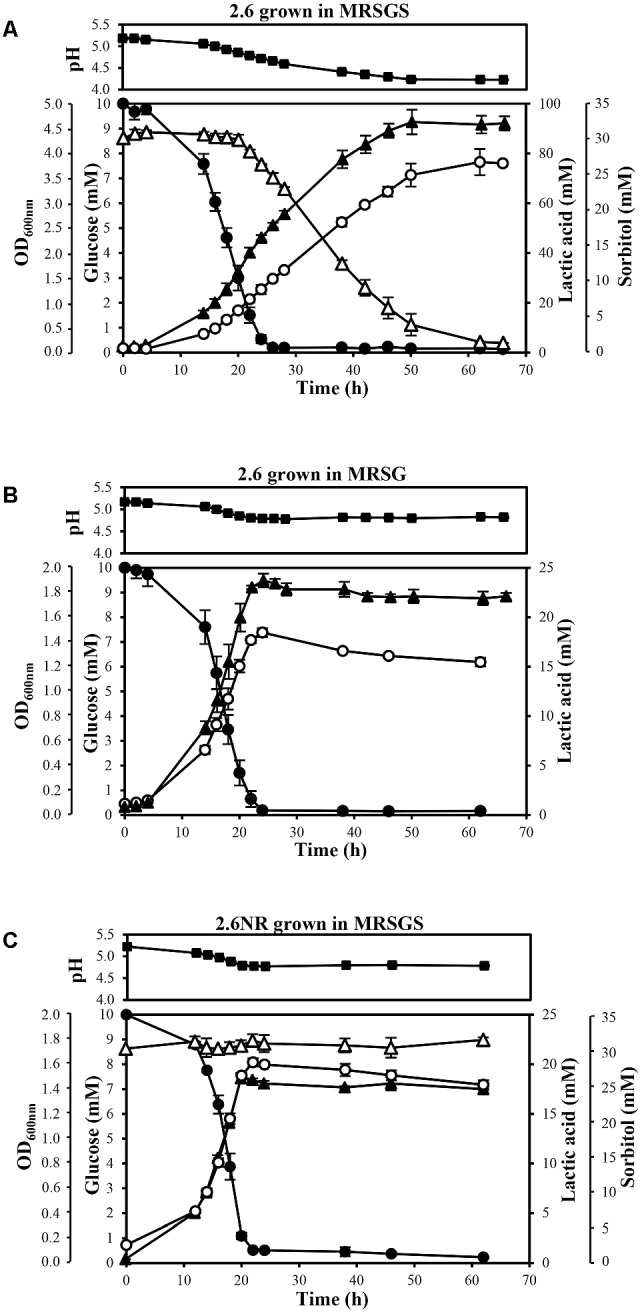
Analysis of metabolism of *P. parvulus* 2.6 **(A,B)** and 2.6NR **(C)** strains. Bacteria were grown in the indicated media. Symbols: OD_600__nm_ (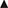), glucose (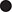), sorbitol (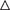), lactic acid (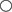) and pH (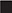). The experiments were performed in triplicate and the mean value and standard deviation is depicted.

*P. parvulus* 2.6 and 2.6NR strains were grown in either MRSG or MRSGS under aerobic conditions (shaking at 180 rpm), at 30°C during 66 h, and samples were taken at the times indicated in **Figure 2** to monitor growth by determination of optical density at 600 nm and of acidification of the media by measuring pH. Also, samples were centrifuged at 16,000 × *g* for 30 min at 4°C, and the levels of glucose, sorbitol, lactic acid and EPS in the supernatants were analyzed. The experiments were performed in triplicate for each strain and in each condition of growth.

#### Analysis of Culture Supernatants by Gas Chromatography-Mass Spectrometry (GC-MS)

The concentration of glucose, sorbitol and lactic acid was determined by GC–MS using myo-inositol as internal standard. For this analysis, *myo*-inositol (100 μg) was first added to aliquots of the bacterial culture supernatants. The mixture was lyophilized and derivatized with 2.5% hydroxylamine chloride in pyridine for 30 min at 70°C, to form the sugar oximes. Afterward, *bis*-trimethylsilyl trifluoroacetamide (BSTFA) was added and samples were incubated for 45 min at 80°C, to form the trimethylsilylated derivatives. Identification and quantification of the compounds were performed by GC–MS on a 7980A-5975C instrument (Agilent, Santa Clara, CA, United States) equipped with a HP-5MS column (30 m × 0.25 mm I.D. × 0.2 μm film thickness) with helium as the carrier gas. Injector and detector were set at 275°C. Samples (1 μL) were injected with a split ratio of 1:50 with a temperature program: 80°C for 4 min, then 15°C min^-1^ to 270°C and finally 30°C min^-1^ to 310°C (2 min). The peaks in the chromatograms corresponding to sugars and lactic acid were identified by their retention times. Quantifications were calculated using the peak areas and the calibration standard curve for each compound.

#### Quantification of the 2-Substituted (1,3)-β-D-Glucan Produced by *P. parvulus*

A competition (ELISA) method for the specific detection of the EPS synthesized by *P. parvulus* 2.6, based on *Streptococcus pneumoniae* serotype 37 antibodies, was performed as previously described (Werning et al., 2014). Briefly, the ELISA assay was carried out in 96-Well Nunc-Immuno MicroWell MaxiSorp plates (Thermo Fisher Scientific), and the EPS of *P. parvulus* 2.6, purified as previously described (Notararigo et al., 2013), was immobilized in each well (62.5 ng per well). Culture supernatants [diluted with phosphate-buffered saline (PBS) pH 7.2 when necessary] were used as competitor for binding to the primary antibody (dilution 1:800 of anti-serotype 37, Statens Serum Institut, Copenhagen, Denmark). Then, primary antibody was conjugated with a secondary antibody, polyclonal Anti-Rabbit IgG alkaline phosphatase (Sigma–Aldrich, Saint Louis, MO, United States) diluted 1:25,000, and finally was revealed with *p*-nitrophenylphosphate in diethanolamine buffer (Sigma–Aldrich). Reaction signals were detected with a microtiter plate reader model 680 (Bio-Rad, Hercules, CA, United States), measuring the OD at 415 nm. Quantification was performed using a standard curve generated by the competition for the primary antibody of serial dilutions of the purified *P. parvulus* 2.6 EPS dissolved in PBS.

### Detection of mCherry Fluorescence in LAB Carrying pRCR16, pRCR17, pRCR18, or pRCR19

To detect the expression levels of the mCherry fluorescent protein, *L. plantarum* Lp90 strains carrying the pRCR derivatives were diluted 1:100 and grown in MRS supplemented with 1% glucose in static mode at 37°C, until mid-exponential phase. Then the cultures were centrifuged at 9,000 × *g* for 10 min at room temperature, and the cells were washed with one volume of PBS pH 7.2 prewarmed at 37°C. Then, the bacteria were resuspended in the same volume of MRS broth supplemented with 1% sorbitol or 1% glucose prewarmed at 37°C. Cultures were incubated at 37°C with agitation of 180 rpm, and samples were taken each hour. Two hundred microliter of all chilled samples were centrifuged at 9,000 × *g* for 10 min at 4 °C and cells were washed once with chilled PBS buffer pH 7.2. Samples were resuspended in 200 μL of PBS buffer pH 7.2 and used to measure the fluorescence levels of mCherry protein in a 96-Well Nunc U96 MicroWell plate (Thermo Fisher Scientific) in a Varioskan Flash equipment (Thermo Fisher Scientific), using 587 and 610 nm wavelengths for excitation and detection of emission, respectively. In addition, appropriate dilutions were prepared to estimate culture biomass by measuring the OD_600__nm_. Three independent trials were performed and the same fresh suspensions, without fixing, were used for phase contrast and fluorescent microscopy analysis with a Leica DM1000 model microscope (Leica Microsystems, Mannheim, Germany) with a light source EL6000 and a filter system TX2 ET for detection of red fluorescence. The microscope was connected to a DFC3000G camera (Leica Microsystems) with a CCD sensor. Image analysis was performed using Leica Application Suite X Software (Leica Microsystems).

To detect the expression of the mCherry fluorescent protein, *L. casei* BL23 strains carrying the pRCR derivatives were grown and processed in the same manner as the *L. plantarum* cultures, except that preinoculum cultures were diluted in MRS supplemented with 1% glucose or 1% sorbitol to an OD_600 nm_ = 0.1 and then were incubated at 37°C with agitation of 180 rpm for 16 h, until they reached early stationary phase. Then, 1 mL of each culture was centrifuged and washed with PBS as above. Samples were concentrated five-fold and used to measure the fluorescence levels and to take fluorescence images as described above.

### Bioinformatic Analysis

The DNA sequence of plasmid pPP1 was analyzed with the programs included in the DNASTAR Lasergene 12 (DNAstar Inc. Madison, WI, United States). Homologies of pPP1 DNA sequences and of its inferred translated products with the NCBI data bases of the National Center for Biotechnology Information (NCBI) were analyzed with the Basic Local Alignment Search Tool (BLAST)^1^. Multiple sequence alignment of genes and proteins were performed with Clustalx 2.1^2^ programs.

Transmembrane helices in GutM were predicted using TMHMM 2.0^3^ (TMpred^4^) programs. Prediction of secondary structures in the gut mRNA was accomplished with the mfold 2.3 program^5^.

## Results

### Analysis of *P. parvulus* Sorbitol Metabolism

Sorbitol could be a substrate for the synthesis of *P. parvulus* 2.6 EPS and analysis of the DNA sequence of the draft genome of this bacterium (Pérez-Ramos et al., 2016) with the BLAST program revealed a putative *gut* operon, that could be involved in transport and catabolism of this compound. Therefore, growth of *P. parvulus* 2.6 and its isogenic EPS-non-producing (non-ropy) 2.6NR strain in MRS (without glucose) and MRSS (medium containing sorbitol) was tested. The 2.6NR strain showed the same poor growth in both media (Supplementary Figure S1). However, the presence of sorbitol in the medium significantly improved the growth of the 2.6 strain (Supplementary Figure S1), reaching a final OD_600__nm_ of 3.0 in MRSS *versus* 0.45 in MRS, indicating that this bacterium was able to utilize sorbitol. Nevertheless, the growth of *P. parvulus* 2.6 in MRSS was very slow and took more than 12 days to reach the final optical density (Supplementary Figure S1). Therefore, in order to improve the growth rate of 2.6 strain, the influence of modifying various parameters in bacterial growth in MRSS was investigated. The best inferred conditions were the usage of a MRSGS containing as carbon sources 10 mM glucose plus 30 mM sorbitol, pH = 5.2, and growth with aeration at 30°C. Thus, these conditions were used to investigate a potential interplay between sorbitol utilization and EPS production by *P. parvulus* 2.6.

A comparative study of the metabolic fluxes of *P. parvulus* strains by analysis of culture supernatants during growth in MRSG or MRSGS corroborated that 2.6, but not 2.6NR, was able to ferment sorbitol (**Figure 2**). Co-metabolism of sorbitol and glucose by the 2.6 strain resulted in an increase of 2.5-fold in the final biomass estimated by the OD_600__nm_ of the cultures. Values of 4.48 ± 0.18 in MRGS (**Figure 2A**) compared to 1.77 ± 0.06 reached in MRSG (**Figure 2B**), the latter being similar to 1.36 ± 0.05 observed for the 2.6NR strain in MRGS (**Figure 2C**). In addition, a prolonged exponential growth phase of the 2.6 strain was observed in the MRSGS medium (50 h *versus* 20 h, **Figures 2A,B**). In the 2.6NR culture supernatants, the initial sorbitol levels (30 mM) remained constant during the entire time period of the assays, revealing that this bacterium was unable to transport sorbitol to the cytosol (**Figure 2C**). Moreover, the analysis of the carbon source consumption by the 2.6 strain showed that glucose started to be transported to the cytosol after 2 h of growth, and upon 26 h of incubation the monosaccharide was undetectable in the culture supernatants (**Figures 2A,B**). Furthermore, only after 20 h of incubation did the 2.6 strain start to internalize the sorbitol and presumably to metabolize it, because the bacterium did not enter into the stationary phase until the sorbitol was consumed (**Figure 2A**). The metabolic activity of the two strains was monitored by detecting the lactic acid production, since it is the main metabolic end-product because pediococci are homofermentative bacteria. The results showed that the 2.6 strain grown in MRSG (**Figure 2B**) and the 2.6NR strain grown in MRSGS (**Figure 2C**) released to the culture media similar amounts of lactic acid, the maximum levels being 18.45 ± 0.45 mM and 20.19 ± 0.42 mM, respectively. By contrast, the 2.6 strain grown in the presence of both carbon sources showed a higher lactic acid production, up to 76.03 ± 0.43 mM (**Figure 2A**). Correlating with these results, the final pH of the 2.6 cultures in MRSG and of the 2.6NR cultures in MRSGS was similar (4.81 ± 0.02 *versus* 4.78 ± 0.02), and higher than that of the 2.6 cultures in MRSGS (4.23 ± 0.02).

**FIGURE 3:**
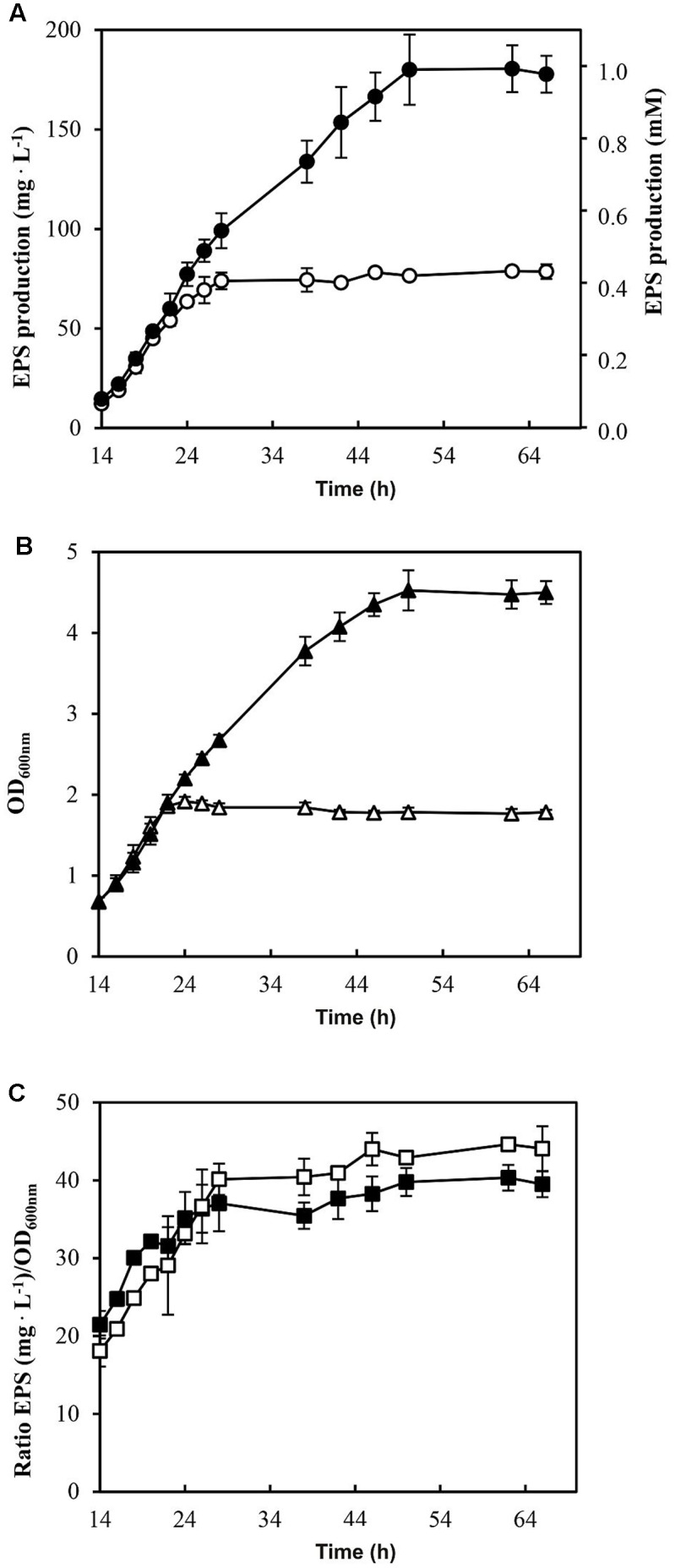
Analysis of EPS production by *P. parvulus* 2.6 in MRSGS (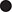), (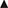) and (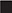) and in MRSG (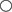), (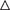) and (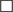). **(A)** Concentration of EPS present in cultures supernatants is depicted. The results were expressed in mg of EPS per L or in mmol of glucose per L. **(B)** The OD_600__nm_ of the cultures is depicted. **(C)** Specific EPS concentration is shown and it was calculated as the ratio EPS concentration/OD_600__nm_. The experiments were performed in triplicate and the mean value and standard deviation is depicted

Furthermore, the EPS production by *P. parvulus* 2.6 in the presence or absence of sorbitol was investigated. Significant EPS levels were detected after 14 h of growth in MRSG and MRSGS media. Therefore, the data depicted in **Figure 3** correspond to those obtained within the 14–62 h incubation period. The results revealed that the bacterium produced EPS during the growth in MRSGS and synthesized higher levels of the polymer in this medium than in MRSG (**Figure 3A**). Thus, after 62 h of growth in MRSG, the 2.6 strain produced 78.6 ± 3.7 mg L^-1^ of EPS, while in MRSGS synthesized 180.5 ± 11.8 mg L^-1^. Additionally, in order to evaluate the specific efficiency of the EPS production depending on the carbon source used, the ratio between EPS concentration and the biomass estimated from the OD_600__nm_ (**Figure 3B**) was calculated (**Figure 3C**). The results showed that irrespectively of the carbon source, the bacteria had almost identical efficiency of EPS production, which increased during the exponential and stationary phases of growth (**Figure 3C**).

### Determination of Genomic Location of the *gut* Operon

*P. parvulus* 2.6 probably carries three natural plasmids, which were previously named pPP1, pPP2 and pPP3 (Werning et al., 2006), and we have identified only three plasmid replication machineries in the *P. parvulus* draft genome (Pérez-Ramos et al., 2016). In addition, the *P. parvulus* 2.6 EPS is synthesized by the GTF glycosyltransferase encoded by the *gtf* gene, which is located in the pPP2 plasmid (Werning et al., 2006). Thus, the 2.6NR strain was generated from 2.6 by pPP2 plasmid curing after treatment with the DNA intercalating agent ethidium bromide and the gyrase inhibitor novobiocin (Fernández et al., 1995).

**FIGURE 4:**
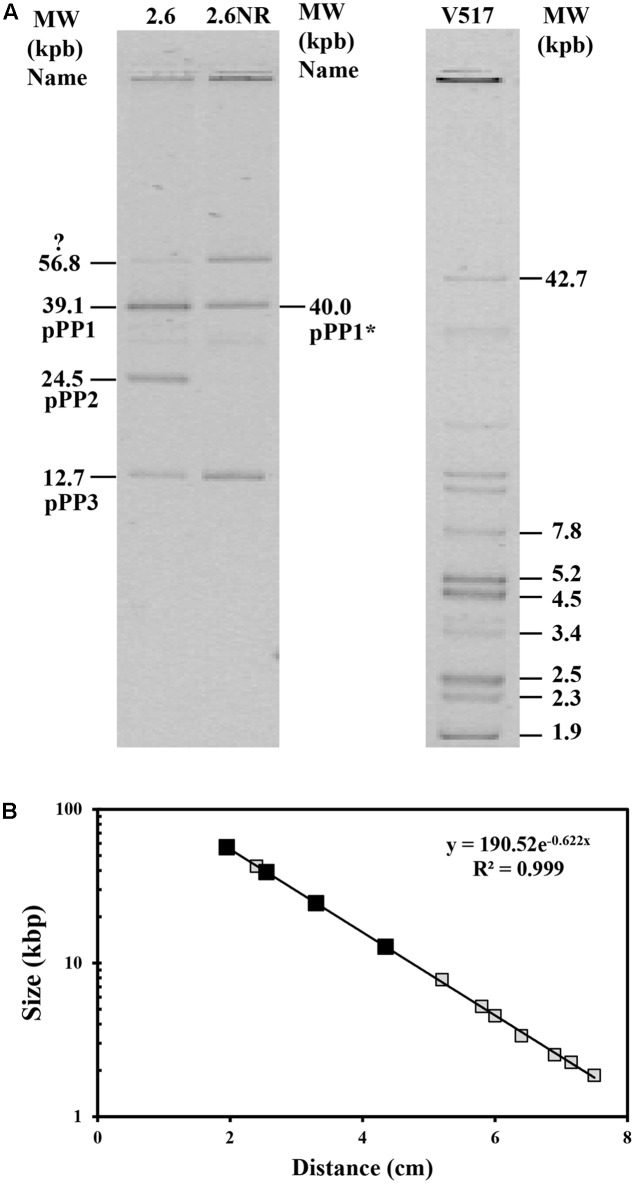
Detection of plasmids of *P. parvulus* 2.6 and 2.6NR strains. Plasmids preparations of *P. parvulus* strains and of *E. coli* V517 were analyzed in a 0.7% agarose gel. **(A)** The image of the gel. The three samples were run in the same gel. In **(B)** the calibration curve for plasmid size determination is depicted. Symbols: plasmids from *E. coli* V517 (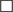), plasmids from *P. parvulus* strains (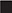).

**FIGURE 5:**
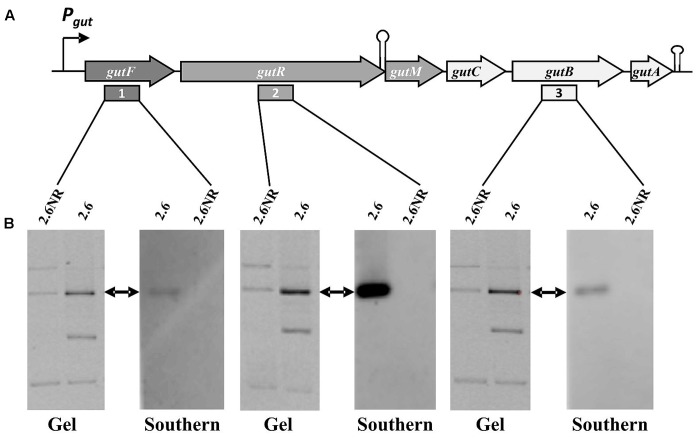
Detection of the *P. parvulus* 2.6 *gut* operon by Southern blot hybridization. Plasmids preparations of the *P. parvulus* strains were fractionated by electrophoresis in agarose gel, transferred to membranes and hybridized for detection of *gutF*, *gutR* and *gutB* with the probes 1, 2, and 3, respectively. **(A)** Physical map of the *gut* operon, including the putative promoter of the *gut* operon (P*_gut_*), the operon genes (*gutF, gutR, gutM gutC, gutB* and *gutA*), a secondary structure including the 3′-end of *gutR* and the 5′-end of *gutM* (coordinates 3483-3512 in GenBank accession No MF766019) as well as a transcriptional terminator (coordinates 6103-6027 in GenBank accession No MF766019) downstream of *gutA.* In addition, location of probes 1, 2, and 3 is indicated. **(B)** Images of the corresponding gels and hybridized membranes. The double headed arrows indicate the position of the hybridized bands in the corresponding gel.

**FIGURE 6:**
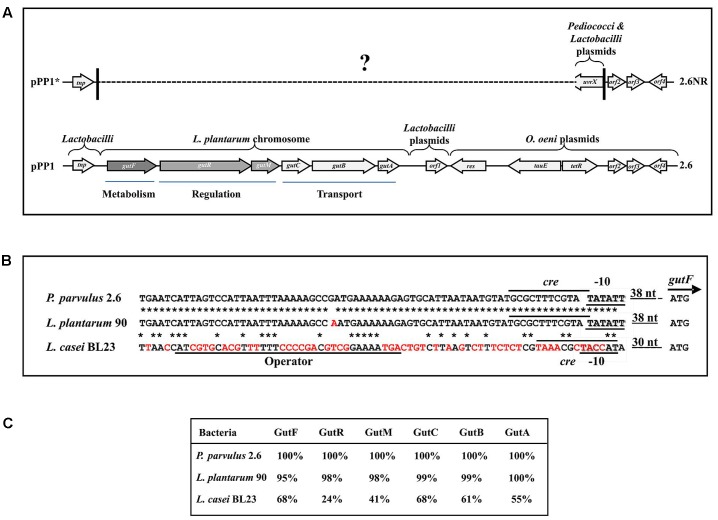
The sorbitol operon and flanking DNA regions of *P. parvulus* 2.6. **(A)** Sequenced regions of pPP1 and pPP1^∗^ plasmids are shown. Genes and ORF are depicted and homologies at the nucleotide level are indicated (see details in the text). ? Indicates unknown sequence. Homology of regulatory genetic regions **(B)** and proteins **(C)** involved in sorbitol utilization between *P. parvulus* 2.6, *L. plantarum* 90 and *L. casei* BL23 is shown. **(B)** DNA sequences of: the putative P*_gut_* promoters (–10 boxes) and the GutR operator of *L. casei* are underlined; the putative *cre* sequences for catabolic repression are overlined; also the start codon (ATG) of *gutF* gene and the nucleotides between this and the –10 boxes are depicted. Red letters indicate mismatches with the *P. parvulus* 2.6 DNA sequence. ^∗^Means identity of nucleotides. **(C)** Identity of the amino acid sequence of the Gut proteins from *L. plantarum* 90 and *L. casei* BL23 with those of *P. parvulus* 2.6 is depicted.

Consequently, given that 2.6NR does not utilize sorbitol, it was feasible that the *gut* operon was encoded by pPP2 and this hypothesis was investigated. First, total plasmidic DNA preparations of the two *Pediococcus* strains were purified by fractionation in a CsCl gradient to eliminate non-supercoiled (open circles and linear) forms of the plasmids. Then, the purified plasmidic DNA preparations were analyzed in an agarose gel (**Figure 4**). Four and three bands were detected, respectively, in preparations of the 2.6 and 2.6NR strains. The sizes of the bands were inferred from their migration using a calibration curve (**Figure 4B**) generated with the plasmids of the *E. coli* V517 strain and are shown in **Figure 4A**. Two of the bands apparently were shared by 2.6 and 2.6NR, and were initially ascribed to the monomeric forms of pPP1 (39.1 kpb in 2.6 and 40.0 kpb in 2.6NR) and pPP3 (12.7 kpb). As expected, pPP2 (24.5 kpb) was not detected in 2.6NR DNA preparations. Moreover, we could not ascribe to any plasmid the band with less mobility and a theoretical molecular weight of 56.8 kbp that was present in DNA preparations of both strains. Quantification of the bands from agarose gels (**Figures 5**, **6**) revealed different proportions of the plasmidic forms in 2.6 (0.3:5.9:2.5:1.0) and 2.6NR (0.8:1.0:0.0:1.0) samples.

The *gut* operon of *P. parvulus* 2.6 (**Figures 5A**, **6A**) is composed of six genes, of which *gutF* encodes a sorbitol-6-phosphate dehydrogenase; *gutRM* encodes two putative regulators; and *gutCBA* encodes the proteins EIIC, EIIBC and EIIA which are components of a phosphoenolpyruvate-dependent sorbitol phosphotransferase system (PTS^gut^). Thus, to detect the location of the *gut* operon, Southern blot hybridization of total plasmidic DNA preparations was performed using as a probe internal regions of *gutF*, *gutR* or *gutB*. One hybridization signal was observed with the three probes at the position of the 39.1 kb pPP1 plasmid in the 2.6 DNA sample (**Figure 5B**). Surprisingly, this plasmid was apparently present in both *P. parvulus* strains, but in the 2.6NR DNA sample no signal was observed. Nevertheless, the results demonstrated that the *gut* operon was not located in the pPP2 plasmid, but rather was carried by the pPP1 plasmid of the 2.6 strain and not of the newly designated pPP1^∗^ plasmid of 2.6NR strain.

### Analysis of Plasmids pPP1 of *P. parvulus* 2.6 and pPP1^∗^ of *P. parvulus* 2.6NR

The results obtained by Southern blot analysis prompted us to obtain further information of pPP1 and pPP1^∗^ plasmids. Thus, the total plasmidic DNA preparation of the 2.6 strain was used as a substrate to confirm the sequence of the gut operon and to determine the unknown nucleotide sequence of the flanking regions (undetected in the draft genome of the bacterium) by the dideoxynucleotide method and with the walking strategy. The sequence of a DNA segment of 11,746 bp (**Figure 6A** and GenBank accession No MF766019) was obtained and its analysis revealed the existence of nine open reading frames (ORF), in addition to the 6 genes (*gutFRMCBA*) of the *gut* operon (**Figure 6A** and Supplementary Table S1). One open reading frame was detected upstream of the gut operon and was designated *tnp*, since its product has 100% identity with a multispecies transposase (Genbank accession No WP_003606336.1) widely distributed in the Lactobacillaceae family. Downstream of the gut operon were detected four ORF named orf1, orf2, orf3 and orf4, which could encode hypothetical proteins conserved in other LAB. In addition, the product of the named *res* gene belongs to the Ser-recombinase superfamily (cl02788) and specifically to the PinE conserved protein domain family (COG1961), showing more than 90% amino acid identity with proteins from oenococci, lactobacilli and pediococci annotated as Pin-related site-specific recombinases/DNA invertases. Also, two divergent genes named *tauE* and *tetR* seem to encode a TauE sulfite exporter which belongs to the TauE conserved domain family (pfam01925) and a transcriptional regulator belonging to the TetR family (domain architecture ID 11442015), and both proteins have more than 95% amino acid identity with their homologues in *Oenoccocus oeni* and lactobacilli.

Based on the DNA sequence of the *gut* operon flanking regions in pPP1, and on the lack of the *gut* operon in 2.6NR, primers were designed and used to try to detect if there exists any identity between pPP1 and pPP1^∗^ by DNA sequencing. Two of these, pPP1^∗^F and pPP1^∗^R, located respectively upstream and downstream of the *gut* operon, provided the desired information (**Figure 6A**). A good chromatogram of the DNA sequencing of pPP1^∗^ using the 2.6NR plasmidic preparation and pPP1^∗^F primer with 100% identity with pPP1 was obtained until nucleotide 156 in the chromatogram (548 nt in Genbank accession No WP_003606336.1), then at least two overlapping sequences were observed (Supplementary Figure S2A), and it was not possible from this point to deduce a further correct DNA sequence. This was not the case when DNA from the 2.6 strain was used as substrate, since a good chromatogram of the pPP1 DNA sequencing was obtained (Supplementary Figure S2B). However, the usage of pPP1^∗^R allowed not only to determine that the homology between pPP1 and pPP1^∗^ starts again at nucleotide 10,021 (in Genbank accession No WP_003606336.1), but also that upstream of this position in pPP1^∗^ there exists a region including a *uvrX* putative gene identical to those of other pediococci (i.e., in pPC892-2 plasmid, Genbank accession No CP021472.1) and *Lactobacilli* (i.e., in pH10 plasmid, Genbank accession No CP002430.1) plasmids, which do not carry *orf2, orf3 and orf4.*

With regard to the *gut* operon of *P. parvulus* 2.6, the identity of the region including the genes and the upstream regulatory regions with the homologues of *L. plantarum* strains was 99% (**Figures 6A,B**) and nucleotides from 694-to 60020 in GenBank accession No MF766019), consequently the amino acid sequence of the Gut proteins of *P. parvulus* showed an identity ranging from 95 to 100%, with those of *L. plantarum* 90 (**Figure 6C**). No significant homology at the DNA sequence level was detected between the characterized operons of *L. casei* and those of *P. parvulus* (**Figure 6B** and results not shown). However, presumably due to convergent evolution, homology ranging from 68 to 24% amino acid identity was detected between the Gut proteins of *P. parvulus* 2.6 and of *L. casei* BL23 (**Figure 6C**).

### Analysis of the *Gut* Operon Regulation

GutR and GutM of *P. parvulus* could be involved in regulation of the *gut* operon expression and upstream of the start codon of *P. parvulus* 2.6 *gutF* gene, a TATAtT sequence was detected that only deviates one nucleotide from the consensus -10 promoter region (**Figure 6B**). Thus, to gain insight into this potential regulation, complementation studies in heterologous LAB hosts able to utilize sorbitol were carried out. First, we cloned independently the putative promoter sequence (designated P*_gut_*) and its upstream region (**Figure 6B**), as well as the transcriptional fusions P*_gut_*-*gutR*, P*_gut_*-*gutM* and P*_gut_*-*gutRM* into the pRCR promoter probe vector (Mohedano et al., 2015) upstream of the *mrfp*, generating the pRCR16, pRCR17, pRCR18 and pRCR19 plasmids, respectively (**Figure 1**). Thus, functionality of the promoter and influence of GutR and GutM could be detected by measuring the levels of fluorescence of the mCherry encoded by the *mrfp* gene. As hosts to perform the studies, we chose: (i) the plasmid free *L. casei* BL23, because its sorbitol utilization and the regulation of its *gut* operon is known (Yebra and Pérez-Martínez, 2002; Nissen et al., 2005; Alcantara et al., 2008) and, (ii) *L. plantarum* 90, because we have previously detected in this bacterium efficient functional expression of mCherry from a pRCR derivative, without problems of plasmid incompatibility and that the copy number of the plasmid was 62 ± 2 molecules per bacterial genome (Russo et al., 2015).

**FIGURE 7:**
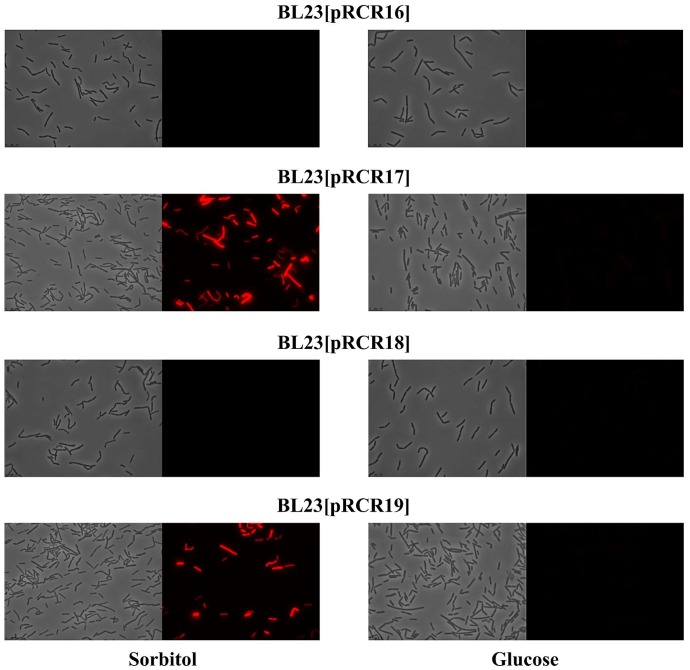
Detection of fluorescence in *L. casei* BL23 strains at stationary phase. Cultures of the indicated strains in MRSS (sorbitol) or MRSG (glucose) were analyzed by phase contrast (left panels) or fluorescence (right panels) microscopy.

The well characterized transcriptional activator GutR of *L. casei* BL23 controls expression of the *gut* operon of this bacteria and its operator site upstream of the P*_gut_* has been identified as well as a catabolite repression element (*cre*) overlapping the -10 region of the promoter (Alcantara et al., 2008) (**Figure 6B**). The *P. parvulus* 2.6 GutR has only a low homology of amino acids (24%) with its homologue of *L. casei*, but like its counterpart belongs to the BglG transcriptional antiterminators family, possesses the PRD domain and the DNA helix turn helix binding domain. Therefore, both proteins could have a similar role. Alignment of the *L. casei* and *P. parvulus* -10 regions revealed that the upstream regulatory regions of BL23 strain has no clear homologs in the 2.6 strain (**Figure 6B**). Consequently, cross talk between transcriptional signals of *P. parvulus* and *L. casei* regulators should not take place, and influence of the pediococcal GutR and GutM in expression of P*_gut_* from the 2.6 strain could be investigated in the BL23 strain without interferences. Thus, the pRCR derivatives were transferred independently to the BL23 strain and the recombinant bacteria were grown in MRS supplemented with either 1% glucose or 1% sorbitol until stationary phase prior to analysis. Examination of the cultures by fluorescent and phase contrast optical microscopy revealed that only bacteria carrying pRCR17 and pRCR19 and grown in presence of sorbitol have fluorescence (**Figure 7**). In addition, fluorescence as well as the optical density of the cultures was measured and the specific fluorescence, referred to the biomass, was calculated. The fluorescence quantification confirmed that the P*_gut_*-*gutRmrfp*, and P*_gut_*-*gutRMmrfp* transcriptional fusions are activated upon growth in the presence of sorbitol (**Table 3**). Thus, these results revealed that expression from the P*_gut_* required the activation by GutR and the presence of sorbitol in the growth medium. Moreover, they indicated that activation by GutR decreased, when GutM was present (5.69 ± 0.44 *versus* 3.58 ± 0.06).

**Table 3:** Heterologous expression of components of the *P. parvulus* 2.6 *gut* operon in *L. casei* BL23 carrying pRCR derivatives plasmids grown in either MRSS or MRSG.

*L. casei* strains	DNA insert in pRCR derivatives	Specific fluorescence^a^	
		MRSS	MRSG
BL23[pRCR16]	P*_gut_*	0.14 ± 0.07	0.14 ± 0.01
BL23[pRCR17]	P*_gut_*-*gutR*	5.69 ± 0.44	0.07 ± 0.09
BL23[pRCR18]	P*_gut_*-*gutM*	0.19 ± 0.06	0.11 ± 0.02
BL23[pRCR19]	P*_gut_*-*gutRM*	3.58 ± 0.06	0.05 ± 0.02

^a^*The specific fluorescence is depicted and it was calculated as the ratio of the detected fluorescence (5×) and the bacterial biomass estimated from the OD_*600 nm*_ of the culture*.

**FIGURE 8:**
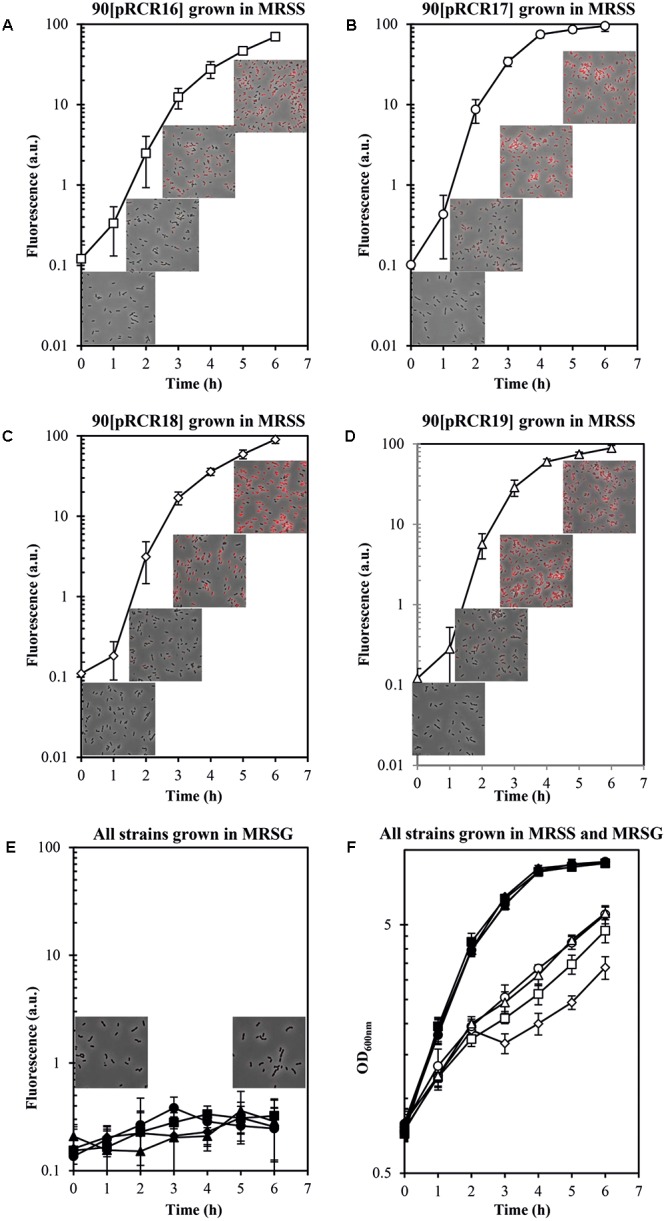
Trans complementation of the sorbitol utilization regulatory machinery in *L. plantarum* 90. Bacteria carrying **(A,E,F)** pRCR16 (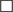, 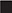), **(B,E,F)** pRCR17 (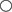, 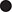), **(C,E,F)** pRCR18 (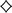, 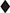) or **(D,E,F)** pRCR19 (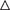, 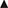) were grown in MRSG and at time 0 were transferred to fresh MRSS (open symbols) or to MRSG (closed symbols). OD_600nm_ and fluorescence of the cultures were monitored every hour. Overlays of images of the cultures taken at time 0 and after 2, 4, and 6 h incubation by phase contrast and fluorescent microscopy are depicted. The experiments were performed in triplicate and the mean value and standard deviation is depicted.

Concerning the *L. plantarum* 90 host, its GutR has 98% homology to that of *P. parvulus* 2.6 (**Figure 6C**) and the DNA sequence of the region located upstream of the two P*_gut_* promoters only differs in one nucleotide (**Figure 6B**). Consequently, both operons must have the same regulatory gene system, which implies that both systems could recognize each other. Thus, a trans-complementation process was expected between the regulatory proteins of Lp90 and the promoter region of 2.6. Therefore, the pRCR derivatives were transferred independently to the 90 strain and, since a cross talk is more complex situation, its comprehension required a more detailed analysis. For this reason, the recombinant bacteria, after growth in MRS supplemented with 1% glucose, were transferred to MRS fresh medium supplemented with either 1% sorbitol or 1% glucose and a time course assay of fluorescence and growth of the cultures was performed. The results revealed that all recombinant strains became fluorescent, when grown in the presence of sorbitol and, with the time of incubation the fluorescence increased (**Figure 8** and **Table 4**). In addition, analysis of the bacterial growth showed that all cultures in MRSG have very similar exponential growth rates (ranging from 0.889 ± 0.059 to 0.803 ± 0.049) and all entered slowly into stationary phase after 2 h of incubation (**Figure 8F** and **Table 4**). Initial transfer of the cultures to MRSS resulted in a similar decrease (around 50%) of the growth rate (values from 0.416 ± 0.045 to 0.495 ± 0.011) during the first 2 h of induction. Then, probably after consumption of the residual intracellular glucose or due to the induction process, bacteria decreased their growth rate to levels ranging from 0.259 ± 0.020 to 0.251 ± 0.034, besides the 90[pRCR18] (GutM overexpressor), that after stalling its growth from 2 h to 3 h incubation time decreased its growth rate to 0.239 ± 0.048, indicating that overexpression of GutM in absence of high levels of GutR has a negative impact for the cells. Furthermore, analysis of the specific levels of fluorescence of the cultures referred to their biomass (**Table 4**) showed different levels for the different fusions (P*_gut_*-*mrfp* < P*_gut_*-*gutRMmrfp* < P*_gut_*-*gutRmrfp <* P*_gut_*-*gutMmrfp*), showing that overexpression of GutM provokes the highest induction of expression from P*_gut_*. In addition, the highest levels were observed after 4 h of induction for cells carrying either pRCR17 (22.38 ± 2.02) or pRCR19 (19.26 ± 2.10) *versus* the end of the incubation (6 h) for cells carrying pRCR16 (14.81 ± 0.66) and pRCR18 (26.83 ± 1.83).

**Table 4:** Heterologous expression of components of *P. parvulus* 2.6 *gut* operon in *L. plantarum* 90 carrying pCRC derivatives plasmids.

*L. plantarum* strains	DNA insert in pRCR derivatives	Medium	Initial growth rate^a^ (μ)	Sorbitol induced growth rate^b^ (μ)	Specific fluorescence^c^				
					2 h	3 h	4 h	5 h	6 h
90[pRCR16]	P*_gut_*	MRSS	0.416 ± 0.045	0.251 ± 0.034	1.48 ± 1.03	5.86 ± 1.43	10.42 ± 1.56	13.44 ± 0.51	14.81 ± 0.66
90[pRCR17]	P*_gut_*-*gutR*	MRSS	0.455 ± 0.065	0.259 ± 0.020	4.42 ± 1.22	13.41 ± 0.90	22.38 ± 2.02	20.30 ± 1.62	17.28 ± 1.65
90[pRCR18]	P*_gut_*-*gutM*	MRSS	0.429 ± 0.025	0.239 ± 0.048	1.62 ± 0.69	10.26 ± 2.30	18.05 ± 2.85	24.26 ± 1.51	26.83 ± 1.83
90[pRCR19]	P*_gut_*-*gutRM*	MRSS	0.495 ± 0.011	0.256 ± 0.016	2.80 ± 0.77	11.74 ± 1.51	19.26 ± 2.10	17.20 ± 1.71	15.98 ± 1.21
90[pRCR16]	P*_gut_*	MRSG	0.889 ± 0.059	ND	0.06 ± 0.03	0.04 ± 0.00	0.04 ± 0.00	0.04 ± 0.01	0.04 ± 0.01
90[pRCR17]	P*_gut_*-*gutR*	MRSG	0.825 ± 0.029	ND	0.07 ± 0.06	0.06 ± 0.02	0.04 ± 0.01	0.03 ± 0.00	0.03 ± 0.02
90[pRCR18]	P*_gut_*-*gutM*	MRSG	0.803 ± 0.049	ND	0.06 ± 0.03	0.03 ± 0.03	0.03 ± 0.01	0.04 ± 0.01	0.03 ± 0.01
90[pRCR19]	P*_gut_*-*gutRM*	MRSG	0.832 ± 0.034	ND	0.04 ± 0.02	0.03 ± 0.02	0.03 ± 0.01	0.04 ± 0.02	0.03 ± 0.02

^a^*Growth rate of the cultures grown in MRSS was calculated from the OD_*600nm*_ obtained during the first 2 h of induction. ^b^Growth rate of the cultures grown in MRSS was calculated from the OD_*600nm*_ obtained during the 2–6 h of induction, besides for 90[pRCR18], that due to the stalling of growth from 2 h to 3 h of incubation the growth rate was calculated from the data obtained from 3 h to 6 h of incubation. ND, the growth rate was not determined because the cultures have entered in the stationary phase of growth. ^c^Specific fluorescence was calculated as the ratio of the detected fluorescence (5x) and the bacterial biomass estimated from the OD_*600*_*nm* of the culture*.

Thus, the results revealed a trans-complementation of the *L. plantarum* regulatory proteins on expression driven from the *P. parvulus* P*_gut_* promoter. Moreover, the results confirmed the role of inducer of GutR as well as requirement of sorbitol for expression from P*_gut_* and support that co-expression of GutR and GutM decrease the activation mediated by GutR.

## Discussion

The overall metabolic results obtained here support that *P. parvulus* is able to synthesize EPS in MRS medium using either glucose or sorbitol as carbon sources. We have previously demonstrated that the 2-substituted (1,3)-β-D-glucan of *P. parvulus* 2.6 is synthesized by the GTF glycosyltransferase utilizing UDP-glucose as substrate (Werning et al., 2014). In addition, Velasco et al. (2007) determined that the 2.6 strain transport the glucose by a PMF-permease and possesses the α-phosphoglucomutase and the UDP-glucose pyrophosphorylase activities responsible for the conversion of glucose-6-P to glucose-1-P and further conversion of this compound to UDP-glucose. Thus, Velasco et al. (2007) showed how the 2.6 strain uses the glucose, not only for the central metabolism, *via* the glycolytic pathway, but also for the secondary metabolism involving a biosynthetic pathway for its EPS synthesis. In addition, the detection of the genetic determinants of sorbitol utilization by the 2.6 strain obtained in this work supports that the bacterium transports sorbitol by a PTS^gut^ system and converts sorbitol-6-P into fructose-6-P by the action of sorbitol-6-P dehydrogenase. Fructose-6-P can be converted to glucose-6-P by a reaction catalyzed by phosphoglucose isomerase, enzymatic activity that was also previously detected in the 2.6 strain (Velasco et al., 2007). Therefore, the 2.6 strain possesses the transport and enzymatic machineries for synthesis of the EPS from sorbitol. In addition, we have detected that aeration of the cultures during the growth improves sorbitol consumption (results not shown). Accordingly, the conversion of sorbitol-6-P into fructose-6-P requires NAD^+^ as an oxidative co-factor to produce NADH (Zarour et al., 2017). Analysis of the draft genome of the 2.6 strain showed the existence of a putative NADH oxidase coding gene. If this enzyme exists, it could unbalance the NAD^+^/NADH equilibrium toward the oxidized form NAD^+^.

The *P. parvulus* 2.6 2-substituted (1,3)-β-D-glucan is composed of molecules of glucose and consequently the EPS concentration can be calculated as molarity of this monosaccharide (see secondary Y axis in **Figure 3A**). This calculation revealed that in both media this bacterium only used a small percentage of the substrate molecules (10 mM glucose plus 30 mM sorbitol in MRSGS or 10 mM glucose in MRSG) for synthesis of EPS (0.99 or 0.45 mM, respectively), whereas more than 90% was utilized in the glycolytic pathway to synthesize pyruvic acid (2 molecules per 1 molecule of substrate) and by action of the lactate dehydrogenase to finally generate lactic acid (1 molecule per 1 molecule of pyruvate, 79 mM or 18 mM). Moreover, the specific quantification method for 2-substituted (1,3)-β-D-glucan used here and the estimation of the specific concentration of EPS synthesized (**Figure 3C**) showed that, using as substrate either glucose or sorbitol, the bacterium synthesizes the same polymer and suggests that with the same efficiency. This was not the case when synthesis of this EPS utilizing fructose was tested, since levels were low compared with that obtained from glucose (Velasco et al., 2007). We have also observed a temporal delay of 2.6 to start to consume sorbitol in MRSGS (**Figure 3A**). This could be due to the existence of a catabolite repression of sorbitol utilization by glucose. Supporting this hypothesis, we have detected a potential *cre* operator (**Figure 6**) for the CcpA, which mediates with HPR this regulation in firmicutes (Deutscher, 2008).

In *P. parvulus* 2.6, the *gut* operon, as in other LAB, constitutes the genetic determinant for sorbitol transport and conversion into fructose-6-P. In addition, we have established here that it is located in a plasmid named pPP1 (**Figure 6**) which is unusual, since the almost identical operon of *L. plantarum* and that of *Lactobacillus pentosus* strain SLC13 (82% homologous, Genbank accession No CP022130.1) as well as the unrelated one from *L. casei* are located in the chromosome. As far as we know, only the location of an unrelated *gut* operon in the megaplasmid pMP118 from *L. salivarius* UCC118 has been previously described (Claesson et al., 2006). A search of the protein data banks revealed that *L. salivarius* 5713 and JCM1046 strains possess, respectively, the pHN3 and pMP1046A megaplasmids which carry *gut* operons homologous to that of pMP118 (Jiménez et al., 2010; Raftis et al., 2014). Flanking the operon two inverted repeat sequences (nucleotides 604-627 and 6612-6635 of Genbank accession No WP_003606336.1) were identified, which are also present at the same relative location in *L. plantarum* strains and at various locations in lactobacilli chromosomes and plasmids (even more than one copy per genome). The upstream region is preceded by a *tnp* gene encoding a putative transposase, which could be responsible for mobilization of the *gut* operon from plasmid to chromosome or *vice versa*.

Lactic acid bacteria are prone to carry more than one compatible plasmid and this facilitates exchange of different regions with physiological significance, that later on can be transferred to other bacteria by plasmid conjugation or mobilization (Cui et al., 2015). Thus, downstream of the *gut* operon of pPP1 there are DNA regions almost identical to that present in plasmids of lactobacilli, which along with *P. parvulus* can be contaminants of alcoholic beverages. Furthermore, the *Oenococcus oeni* pOENI-1 and pOENI-1v2 plasmids (Favier et al., 2012) and pPP1 carry a region containing among others the *res, tauE* and *tetR* genes. The putative TauE sulfite exporter is possibly involved in adaptation to stress conditions during alcoholic beverage production (Favier et al., 2012). Thus, the recombinase or invertase site specific Res could be responsible for a mobilization of an element composed of a truncated *res, tauE* and *tetR* to a stable location, since at the 3′-end region of *res* and downstream of *tetR* unit exist inverted complementary sequences 5′-TTTTAAAGC-3′ and 5′-GCTTTAAAA-3′ (nucleotides 7774–7778 and 10021–10029 of Genbank accession No WP_003606336.1).

Another instance of plasmids rearrangement in *P. parvulus* is that which generated the profile and DNA sequence of 2.6NR strain plasmids (**Figure 6**). The initial isolate of 2.6NR strain generated in the Basque country University (BCU, Spain) and described in Fernández et al. (1995), kindly provided by Dr. Maria Teresa Dueñas (BCU) was studied in this work. Thus, the changes in plasmid cassettes were not produced in our laboratory, and presumably they took place upon treatment of 2.6 strain with ethidium bromide and novobiocin and was selected for the loss of the ropy phenotype. Thus, it is feasible that a formation of a co-integrate of pPP1 with other plasmid, may be pPP2, took place and convergent replication from two origins prompted to a deletion of one of the replicons, may be the pPP2, since its loss was envisaged, to generate pPP1^∗^.

**FIGURE 9:**
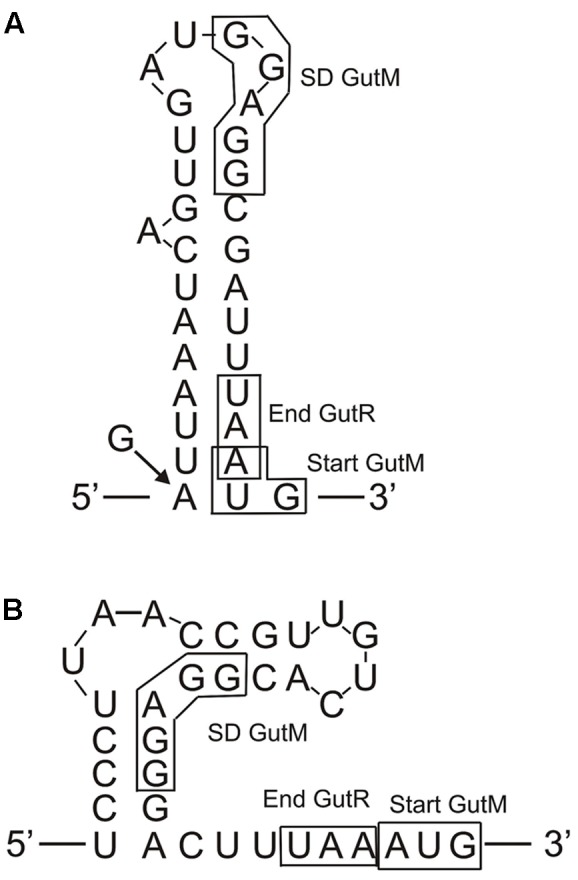
Predicted folding of the *gut* mRNA region including the 3′-end of *gutR* and the 5′-end of *gutM*. **(A)** Secondary structure of the wild-type *P. parvulus* 2.6 and *L. plantarum* 90 *gut* operons. The arrow and the G indicate that in the insert of pRCR18 G substitute to the A in the indicated position. **(B)** Secondary structure of the *L. casei* BL23 *gut* operon.

Concerning the regulation of expression of the *gut* operon, the overall results showed that it is repressed in the absence of sorbitol in the growth medium and that the *P. parvulus* GutR is an activator like the *L. casei* BL23 regulator (Alcantara et al., 2008). In this system, it has been proposed that GutM is involved in the activation, since a decreased expression of the *gut* operon was detected in a GutM deficient mutant (Alcantara et al., 2008). Furthermore, the complementation studies in *L. plantarum* 90 performed here showed a heterologous regulation of gene expression from the pediococcal P*_gut_* promoter by the GutR from *Lactobacillus*, and a positive effect when only the pediococcal GutM was overexpressed (**Figure 8** and **Table 4**). Thus, these results suggest that a protein-protein interaction between the *P. parvulus* GutM and the *L. plantarum* GutR could potentiate the activation of the P*_gut_* promoter, since, *P. parvulus* 2.6 GutR and GutM have 98% identity with their homologues of *L. plantarum* 90. In addition, either in *L. casei* BL23 and in *L. plantarum* a decrease of expression from P*_gut_* was observed when GutM was overexpressed in combination with GutR (**Figure 8** and **Tables 3**, **4**). This prompted us to analyze the genetic environment of *gutR* and *gutM.* An overlapping of the last nucleotide of the termination codon (TAA) of *gutR* and the first nucleotide of the start codon (ATG) of *gutM* was detected in *P. parvulus* 2.6 and *L. plantarum* 90 genomes. This indicated that post-transcriptional regulation of the *gut* operon could exist in this bacterium. For this reason, the secondary structure of the region surrounding the overlapping in the *gut* mRNA was folded with the Mfol program (**Figure 9A**). A secondary stem-loop structure was predicted with a ΔG = -5.6 kcal mol^-1^, the ribosomal binding site (RBS) of *gutM* (5′-GGAGG-3′) was located at the loop and partially blocked in the stem of the structure. Thus, even though the sequence of the RBS of *gutM* indicates a high efficiency of utilization for the ribosome, the initiation of translation of *gutM* could be partially impaired by the partial RBS blockage, which would be released by the opening provoked by the passage of the ribosomes translating *gutR*. In addition, the overlapping of *gutR* and *gutM* is located at the end of the stem of the structure. Thus, two post-transcriptional regulations could take place: (i) translation of *gutR* can act by favoring translation of *gutM* by exposition of its RBS and (ii) a -1 frameshift (Atkins et al., 2016) could happen at the TAA termination codon of *gutR* and ribosomes translating this could step back one nucleotide and upon charging the corresponding tRNA read the Leu (TTA) codon and continue translating *gutM.* In this way a fused peptide GutR-M could be synthesized. The same structure could be formed in the transcript encoded by the plasmid pRCR19 with a ΔG = -5.9 kcal mol^-1^, containing *gutRM* and which could be a substrate for the two proposed post-transcriptional regulations. Furthermore, the DNA fragment cloned in pRCR18, lacks most of the *gutR* gene but still retains some of the 3′-end region of this gene and the encoded mRNA can form a secondary structure almost identical to the wild-type structure (with only a change of A-U by G-U pairing at the end of the stem, **Figure 9**). Thus in bacteria carrying pRCR18 partial blockage of the RBS could take place, but synthesis of GutR-M could not occur. This could explain the antagonistic effect of overexpression of *gutM* from pRCR18 (increase of expression from P*_gut_*) and pRCR19 (decrease of expression from P*_gut_*), if GutR-M exists and has a role.

Prediction of transmembrane regions in the regulatory proteins with the TM-Pred revealed that GutR is a soluble protein and that the first amino acids from 1 to 21 of GutM constituted a transmembrane region also predicted for the GutR-M fused polypeptide. This fused polypeptide could provide an efficient anchoring of the regulator to the membrane bringing it close to the PTS^gut^ system facilitating the phosphorylation of GutR and resulting in the physiological optimal expression of the operon. This generation of a fused polypeptide could also take place in *L. plantarum* but does not seems to occur in *L. casei*, since in this bacterium the TAA translational stop codon of GutR and the ATG start codon of GutM are adjacent and not overlapped (**Figure 9**). However, the *L. casei gut* transcript can form a secondary structure with a ΔG = -9.8 kcal mol^-1^ which could block the RBS of *gutM* gene, couple translation of GutR and GutM could take place, and protein-protein interaction could be responsible for higher activation of the system at the beginning of the induction process. Our results indicate that high levels of GutM synthesized from a multicopy plasmid have a deleterious effect for the bacteria (**Figure 8**) and probably the proposed models of posttranscriptional regulation are designed to have the right concentration of regulatory proteins. Nevertheless, further experiments are required to pinpoint the role of GutM and of the putative GutR-M polypeptide of *P. parvulus*.

## Author Contributions

AP-R contributed to all parts of the experimental work and wrote a draft of the manuscript. MW performed the initial detection of sorbitol utilization and characterization of *gut* genes. AP contributed to the characterization of the sorbitol metabolism. PR participated in the elaboration of the manuscript and analysis of the DNA sequences. GS contributed to the design and analysis of the experimental work involving characterization of regulation of *gut* operon expression. MM contributed to the design of strategies to determine trans complementation of the *gut* operon and corrected the manuscript. PL participated in study conception, data interpretation and generated the final version of the manuscript. All authors have read and approved the final manuscript.

## Conflict of Interest Statement

The authors declare that the research was conducted in the absence of any commercial or financial relationships that could be construed as a potential conflict of interest.

## Footnotes

1 https://blast.ncbi.nlm.nih.gov/Blast.cgi

2 http://www.ebi.ac.uk/Tools/msa/clustalw2/

3 http://www.cbs.dtu.dk/services/TMHMM-2.0/

4 http://embnet.vital-it.ch/software/TMPRED_form.html

5 http://unafold.rna.albany.edu/?q=mfold/RNA-Folding-Form
