# Supplementary material for: Characterization of the Sorbitol Utilization Cluster of the Probiotic Pediococcus parvulus 2.6: Genetic, Functional and Complementation Studies in Heterologous Hosts
Source: Front Microbiol. 2017 Dec 5;8:2393. doi: 10.3389/fmicb.2017.02393 (PMC5723342; doi:10.3389/fmicb.2017.02393)
Supplement: Supplementary file 1 [file Data_Sheet_1.DOC]

Supplementary Material

**Molecular and physiological characterization of sorbitol utilization by the probiotic *Pediococcus parvulus* 2.6**

***Pérez-Ramos A.^1^, Werning, M.L.^1,2^, Prieto A.^1^, Russo P.^3^, Spano G.^3^, Mohedano M.L.^1^, López P.^1*^***

^1^Biological Research Center (CIB), CSIC, Madrid, Spain

^2^Center of Research and Transfer of Catamarca (CITCA), CONICET, Catamarca, Argentina

^3^Department of Agricultural, Food and Environmental Sciences, University of Foggia, Foggia, Italy

*** Correspondence:**

Dr. Paloma López
[plg@cib.csic.es](mailto:plg@cib.csic.es)


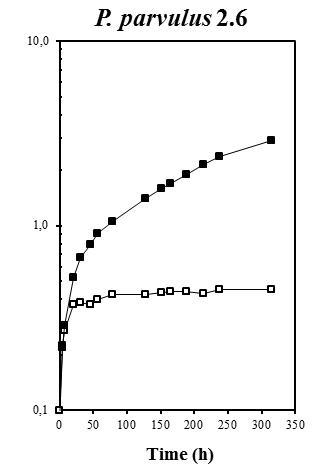

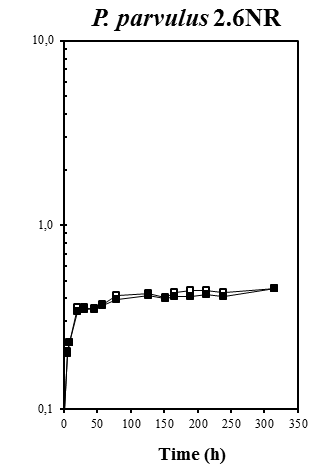


10.0

10.0

1.0

1.0

0.1

0.1

**OD_600nm_**

**OD_600nm_**

**Figure S1. Influence of sorbitol in growth of *P. parvulus* 2.6 and 2.6NR***.* The bacteria were grown in either MRS (□) or MRSS (■) media.

**Figure S2. Chromatograms of the rearrangement of pPP1 to generate pPP1*.** DNA sequence was performed with the dideoxinucleotide method by using total plasmidic preparation of the indicated strains and the pPP1*F as substrates. * indicates mismatch between the two DNA sequences (A and B). The vertical arrows indicates nucleotides from which the two DNA sequences differ.

**Table S1. Genes and ORF present in plasmid pPP1 of *P. parvulus*** 2.6

| **Gene** | | | | **Protein** | |
| --- | --- | --- | --- | --- | --- |
| **Name** | **Start** | **Stop** | **Name** | **No of amino acids** | **Function** |
| *tnp* | 50 | 433 | Tnp | 127 | Multispecie transposase |
| *gutF* | 808 | 1611 | GutF | 267 | Sorbitol-6-phospate dehydrogenase |
| *gutR* | 1645 | 3510 | GutR | 621 | Sorbitol operon transcriptional regulator |
| *gutM* | 3510 | 4016 | GutM | 168 | Sorbitol operon activator |
| *gutC* | 4021 | 4572 | GutC | 183 | Sorbitol PTS, EIIC component |
| *gutB* | 4596 | 5615 | GutB | 339 | Sorbitol PTS, EIIBC component |
| *gutA* | 5643 | 6020 | GutA | 125 | Sorbitol PTS, EIIA component |
| *orf1* | 6815 | 7174 | ORF1 | 119 | Hypotetical protein |
| *res* | 7845 | 7258 | Res | 195 | PIN-related DNA recombinase/invertase |
| *tauE* | 9239 | 8397 | TauE | 280 | Putative permease, Sulfite exporter TauE/SafE |
| *tetR* | 9346 | 9930 | TetR | 194 | TetR family transcriptional regulator |
| *Orf2* | 10422 | 10664 | ORF2 | 80 | Hypotetical protein |
| *Orf3* | 10726 | 11043 | ORF3 | 105 | Hypotetical protein |
| *Orf4* | 11460 | 11161 | ORF4 | 99 | Hypotetical protein |
